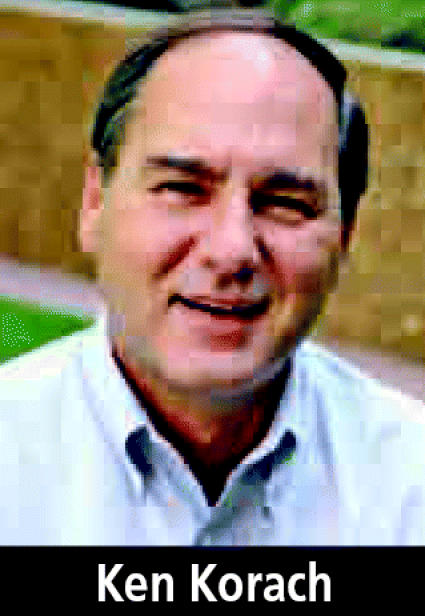# Editorial Note: New Interim Editor-in-Chief

**Published:** 2007-01

With this issue, Kenneth S. Korach becomes interim editor-in-chief of *EHP*. Korach is director of the Environmental Diseases and Medicine Program, chief of the Laboratory of Reproductive and Developmental Toxicology, and chief of the Receptor Biology Section at the NIEHS. He received his PhD in endocrinology from the Medical College of Georgia in 1974. Korach’s areas of research include the basic mechanisms of estrogen hormone action in reproductive tract and bone tissues, with an application toward understanding how hormonally active environmental estrogens influence physiological processes. He is the past editor-in-chief of *Endocrinology*, the flagship journal of the American Endocrine Society. Korach will guide *EHP* until a permanent editor-in-chief is selected.

## Figures and Tables

**Figure:**